# Reactivation of Motor-Related Gamma Activity in Human NREM Sleep

**DOI:** 10.3389/fnins.2020.00449

**Published:** 2020-05-12

**Authors:** Jean-Baptiste Eichenlaub, Siddharth Biswal, Noam Peled, Nicole Rivilis, Alexandra J. Golby, Jong Woo Lee, M. Brandon Westover, Eric Halgren, Sydney S. Cash

**Affiliations:** ^1^Department of Neurology, Massachusetts General Hospital, Harvard Medical School, Boston, MA, United States; ^2^Department of Radiology, Massachusetts General Hospital, Harvard Medical School, Boston, MA, United States; ^3^Department of Neurosurgery, Brigham and Women’s Hospital, Harvard Medical School, Boston, MA, United States; ^4^Department of Neurology, Brigham and Women’s Hospital, Harvard Medical School, Boston, MA, United States; ^5^Departments of Radiology and Neuroscience, Kavli Institute for Brain and Mind, University of California, San Diego, San Diego, CA, United States

**Keywords:** memory reactivation, human sleep, NREM sleep, intracranial recordings, machine-learning

## Abstract

Models of memory consolidation posit a central role for reactivation of brain activity patterns during sleep, especially in non-Rapid Eye Movement (NREM) sleep. While such “replay” of recent waking experiences has been well-demonstrated in rodents, electrophysiological evidence of reactivation in human sleep is still largely lacking. In this intracranial study in patients with epilepsy (*N* = 9) we explored the spontaneous electroencephalographic reactivation during sleep of spatial patterns of brain activity evoked by motor learning. We first extracted the gamma-band (60–140 Hz) patterns underlying finger movements during a tapping task and underlying no-movement during a short rest period just prior to the task, and trained a binary classifier to discriminate between motor movements vs. rest. We then used the trained model on NREM sleep data immediately after the task and on NREM sleep during a control sleep period preceding the task. Compared with the control sleep period, we found, at the subject level, an increase in the detection rate of motor-related patterns during sleep following the task, but without association with performance changes. These data provide electrophysiological support for the reoccurrence in NREM sleep of the neural activity related to previous waking experience, i.e. that a basic tenet of the reactivation theory does occur in human sleep.

## Introduction

Numerous empirical studies (Plihal and Born, 1997; Walker et al., 2002) and theoretical accounts (Diekelmann and Born, 2010; Stickgold and Walker, 2013) highlight the reprocessing of memories from recent waking experiences by the sleeping brain. This sleep-dependent processing of newly acquired memories refers to multiple processes as reflected by its behavioral correlates, including memory enhancement (e.g. Walker et al., 2002), integration (e.g. Tamminen et al., 2010), selection (e.g. Wilhelm et al., 2011) or stabilization (e.g. Nettersheim et al., 2015).

At the brain level, a fundamental question concerns the neural mechanisms underlying such processing. Thanks to the monitoring of the spike activity from multiple neurons, neural replay has been observed in non-human animals (see, e.g. Pavlides and Winson, 1989; Wilson and McNaughton, 1994; Lee and Wilson, 2002; Ji and Wilson, 2007; Gulati et al., 2014). Neural replay refers to the spontaneous (as opposed to externally cued) recapitulation during rest, of the ensemble firing patterns observed during prior waking experience, and has been proposed as a potential mechanism underlying the offline processing of recent memories (for reviews, see Schwindel and McNaughton, 2011; Abel et al., 2013; Atherton et al., 2015).

In humans, indirect measure of neural replay, often termed neural reactivation, has been demonstrated, especially using positron emission tomography (PET) or functional magnetic resonance imaging (fMRI) (e.g. Maquet et al., 2000; Peigneux et al., 2004; Deuker et al., 2013; Schapiro et al., 2018). In a seminal series of PET studies, it has been shown that several brain areas activated during the execution of a motor task were significantly more active during rapid-eye movement (REM) sleep in subjects previously trained on the task than in those who were not (Maquet et al., 2000), and that the level of hippocampal reactivation during non-REM (NREM) sleep was correlated with performance improvement in a navigation task (Peigneux et al., 2004). More recently, fMRI studies have reported reactivation of patterns specific to individual learning experiences in both wake and sleep following a paired-associate learning task (Deuker et al., 2013; Staresina et al., 2013; Tambini and Davachi, 2013; Schapiro et al., 2018), as well as motor-related reactivation in NREM sleep following a motor learning task (Fogel et al., 2017; Vahdat et al., 2017).

Other work exploited electroencephalography (EEG) recordings to provide more direct, electrophysiological evidence of (spontaneous) reactivation in humans (Jiang et al., 2017; Schönauer et al., 2017; Zhang et al., 2018). Using pattern analysis applied on scalp EEG, Schönauer et al. (2017) showed that human sleep EEG contains features specific to the type of visual task previously experienced. Thanks to the intracranial EEG (iEEG) recordings in patients undergoing clinical monitoring for epilepsy, Jiang et al. (2017) reported the reactivation in NREM sleep of spatiotemporal sequences of gamma activity peaks that were identified in previous waking periods. Similarly, Zhang et al. (2018) reported awake and NREM sleep reactivation of item-specific gamma activation patterns that were initially identified in a visual memory task. However, electrophysiological evidence of spontaneous reactivation in humans is still sparsely documented, especially in the motor learning domain.

The neural activity in the high-frequency band (above ∼50 Hz, i.e. gamma-band) is thought to be an index of a multitude of cognitive processes, including memory and motor functions (for reviews, see Lachaux et al., 2012; Johnson and Knight, 2015). In addition, gamma activity is correlated with local spiking activity (Manning et al., 2009), and hemodynamic signals (Nir et al., 2007), and, as described above, have been recently related to reactivation mechanisms in human sleep (Jiang et al., 2017; Zhang et al., 2018). Based on these results, the present study aimed to explore the spontaneous (i.e. non-externally cued) reactivation during sleep of gamma-band patterns underlying previous motor learning in human iEEG recordings. We hypothesized that the gamma-band activity patterns underlying finger-movements re-occurs more often during sleep following the task than during an earlier control sleep period. First, we trained a support vector machine model (binary classification: “motor” vs. “rest”) to extract the spatial gamma-band patterns underlying finger movements during a tapping task and underlying no-movement during a short rest period just prior to the task. Then we applied the trained model on the “unlabeled” NREM sleep data immediately after the task and on control data from a sleep period preceding the task. We observed, at the single subject level, an increase in the proportion of windows labeled as motor activity during sleep following the task, showing that gamma-band patterns recorded during motor learning re-occurred during subsequent sleep. These data provide electrophysiological evidence for the occurrence of reactivation mechanisms in human sleep and highlight the role of gamma activity in such processing.

## Materials and Methods

### Subjects

The data were collected from January 2014 to September 2015 at Massachusetts General Hospital and Brigham and Women’s Hospital in fifteen patients with long-standing pharmaco-resistant epilepsy. The patients gave informed consent, and the research protocol was approved by the local Institutional Review Board (Partners IRB).

Intracranial EEG (iEEG) recordings were made over the course of clinical monitoring for spontaneous seizures. Participants were implanted with multi-lead depth electrodes (i.e. stereotactic-EEG, sEEG) or subdural grid electrode arrays (i.e. Electrocorticography, ECoG) to confirm the hypothesized seizure focus and locate epileptogenic tissue in relation to essential cortex, thus directing surgical treatment. The decision to implant, the electrode targets and the duration of implantation were made on clinical grounds without reference to this research.

From this dataset, we studied nine patients (age range: 17–59 years; 4 females; Supplementary Table 1) who satisfied the following inclusion and exclusion criteria. Inclusion criteria included a post-learning sleep session (between the end of the learning and start of the retest sessions) lasting 2 h maximum and containing at least 1 epoch of NREM (N2 or N3) sleep. The exclusion criteria included the occurrence of identified electroclinical or electrographic seizures 2 h before and after the experiment (including sleep periods). These criteria were selected to obtain a reasonable degree of uniformity across participants and avoid potential confounds. Participants were all right-handed, with intelligence in the normal range.

### Electrodes and Recordings

sEEG subjects (*N* = 5, Supplementary Table 1) had 10 to 12 probes implanted orthogonally to the midsagittal plane. Probes had a diameter of 1.2 mm and consisted of 6 or 8 platinum/iridium-contact leads 2.4 mm long at 10 mm centers. In one sEEG participant, two linear ECoG arrays (4 × 1 contacts-arrays) completed the spatial coverage but were not considered in the current analysis. ECoG subjects (*N* = 4) received ECoG contacts that were 3 mm platinum/iridium disks spaced 10 mm center-to-center, embedded in a main 8 × 8 (*N* = 2), 6 × 8 (*N* = 1) or 4 × 7 (*N* = 1) grid array. In some ECoG participants, additional arrays/probes completed the spatial coverage but were not considered in the current analysis.

Recordings were made using a research-dedicated system (Neural Signal Processor, Blackrock Microsystems, Salt Lake City, UT, United States) with a 2000 Hz sampling rate or with a clinical EEG monitoring system (XLTEK, Natus Medical Inc., Peasanton, CA, United States) with a 250 Hz sampling rate. At the time of acquisition, depth recordings were referenced to scalp EEG and grid recordings were referenced to epidural electrodes facing away from the cortex.

### Electrode Coordinates and Labeling Algorithm

Electrode coordinates were computed using a volumetric image coregistration procedure. Using Freesurfer scripts^1^, the preoperative T1-weighted MRI (showing the brain anatomy) was aligned with a postoperative CT (showing electrode locations). Electrode coordinates were manually determined from the CT and placed into the native space. To take into account misalignment between MRI and CT scans due to craniotomy in patients implanted with subdural grid electrode arrays, we applied an energy-minimization procedure to project grid contacts onto the cortical surface (Dykstra et al., 2012).

An electrode labeling algorithm^2^ was used to assign a labeled brain region to each electrode using the Freesurfer’s DKT 40 atlas (Klein and Tourville, 2012) in combination with a subcortical mapping enabled through Freesurfer. Succinctly, ELA identifies the probability that a given labeled region of the brain is a source of a given electrode. It operates with the assumption that this probability is estimated based on the Euclidean distance between the brain label and the electrode; both being defined by means of the “brain label” and “electrode” voxels, respectively. Brain label voxels are all voxels where at least one of the label vertices is positioned inside the voxel volume as mapped in the structural MRI. Electrode voxels are the voxels where the distance from the center to the electrode is smaller than a given threshold, Dc, circumscribing a “cloud” around the electrode. For depth electrode, the cloud was cigar-shaped with length 4.0 mm and diameter 3.0 mm, and the center was localized in between each two adjoining electrodes on the same lead since a bipolar montage was applied. For grid electrode, the cloud was a 2D sphere on the cortical surface with a diameter of 3.0 mm. To estimate the probability, the ELA counts the number of intersecting voxels between the electrode voxel “cloud” and the label voxels and divides that count by the number of the electrode voxels i.e. the probability that the given electrode is getting a signal from a given label. In the case where none of the labels voxels intersects with the electrode voxels for a given electrode, Dc is expanded by ΔDc (depth electrode: length was expanded of 1.0 mm and diameter of 0.5 mm; grid electrode: diameter was expanded of 0.5 mm). This expansion continued until a detectable intersection between a label voxels and the electrode voxels is found. For each electrode, the brain label with the highest probability was finally extracted and used for further analysis.

### Motor Learning Paradigm

Presentation Software (Neurobehavioral System, Berkeley, CA, United States) was used for the stimulus delivery and motor response recording. Participants were seated comfortably on their hospital bed in front of a computer monitor, and the instruction was displayed on the screen at the beginning of the task. The finger tapping task required participants to press four numeric keys on a standard computer keyboard with four different fingers, repeating a unique sequence of six digits (e.g. 4-2-1-3-4-1) “as quickly and accurately as possible” for a period of 30 s. The sequence was continuously displayed on the screen to minimize any working memory component of the task. During each repetition, each key press produced a gray dot below the ongoing number, forming a row from left to right, to inform participants about the ongoing sequence while not providing accuracy feedback. Participants were instructed to keep going if they realized an error was made.

The learning session consisted of ten 30 s blocks with 30 s breaks between blocks. After each 30 s break, the instruction reminded on the screen (“Repeat the sequence displayed on the screen as quickly and accurately as possible – you have 30 s”) and the participants were asked to press enter when ready to start the next 30 s-long tapping block. The retest session (e.g. after the nap period) consisted of at least three 30 s blocks (some participants performed up to 6 30 s blocks but only the first three 30 s-trials were considered during retest). Motor performance was calculated as the time (in seconds) to correctly play the 6-digit sequence. To exclude extremely slow sequences, repetitions lasting more than mean +3 (standard deviation, SD) were excluded. On average, the percentage of excluded sequences was 1.92% (range: 1.16–3.61%) and the number of accepted sequences was 88.11 (range: 56–166). Each repetition was finally divided by the mean from all accepted repetitions. This yields performance values expressed relative to a “baseline level,” thus facilitating comparison between participants.

### Procedure

The experiment was performed during the day in accordance with participant and clinical team schedules and was set up to last ∼120 min in total (mean = 104.6, SD = 22.5 min; from the initial rest session to the final retest). The participants were initially briefed about the task. They were aware that the study was focused on sleep and that they would be retested after the nap session. During the initial rest session that immediately preceded the learning task, participants were instructed to stay relaxed with eyes closed for ∼4 min (mean = 240.0, SD = 52.0 s; except participant P#1 who stayed eyes opened and fixed on a cross on the screen). Following that rest session, the participants performed the motor learning task (learning session). They were then allowed to sleep during a nap session (post-learning sleep, “sleepPost”) before being retested on the same task (retest session).

**FIGURE 1:**
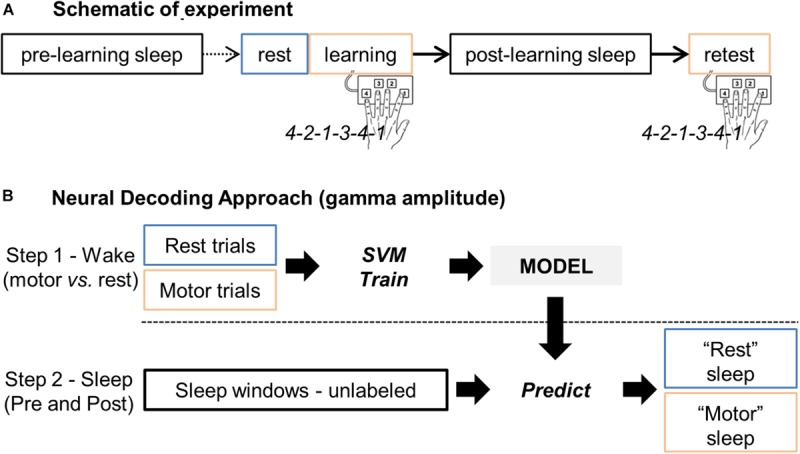
**(A)** Schematic representation of the experiment, including the rest, learning and retest sessions, and the two sleep periods before (pre-learning sleep or “sleepPre”) and after (post-learning sleep or “sleepPost”) learning, respectively. **(B)** Main steps of the neural decoding approach. Gamma band activity during rest and finger tapping were first used as features (i.e. predictors) to train a support vector machine (SVM) model for two-class classification i.e. “motor” vs. “rest” classes. The trained model was then applied to the unlabeled sleep samples: the gamma band activity during sleep was processed by the model to return for each windowed sleep trial a class label (motor- or rest-vote). Finally, the proportion of sleep windows classified as “motor” (i.e. the proportion of motor-votes) was compared between the two sleep periods (sleepPre vs. sleepPost).

Based on the hypnogram during this post-learning sleep and by going backward in time, a control sleep period prior to the motor learning (“sleepPre”) was identified so that the 2 sleep periods did not differ in terms of their sleep characteristics. This approach permitted close correlation of the amount of elapsed time between the task and the 2 sleep periods, and also minimization of potential brain signal nonstationarity effects. Figure 1A summarizes the procedure.

### Sleep-Stage Scoring

Stages of vigilance (wake, NREM sleep, REM sleep) were scored visually (JBE) and in 30-s epochs using Python 3.5 with phypno^3^. The scoring was performed from sEEG or ECoG electrodes (in combination with scalp electrodes when available) and followed as closely as possible the standard sleep stage classification (Iber et al., 2007). Electrooculography (EOG), electromyography (EMG) and digital video recording completed the sleep scoring apparatus when available.

### Data Preprocessing

Data analysis was performed using custom analysis code in Matlab (MathWorks Inc) and using the Fieldtrip toolbox (Oostenveld et al., 2011), an open-source software implemented in Matlab.

#### EEG Preprocessing

Each iEEG segment (sleepPre, rest/learning, sleepPost, and retest) was preprocessed independently for computational purposes. Channels which were non- or mal-functioning were first visually identified and excluded (mean = 4.0, SD = 3.5 channels per participant). The data were de-meaned and the line-noise removed using band-stop filters ([58–62, 118–122], zero-phase forward and reverse Butterworth filter, 4th order, implemented in Fieldtrip). The data were then re-referenced with respect to their direct neighbors (bipolar montage, depth electrodes) or using a common average reference (grids electrodes).

Pathological channels contaminated by interictal epileptiform discharges (IEDs) were identified in an automatic manner by using an IED detection algorithm (Janca et al., 2015; version v21, default settings except -h 60). The algorithm adaptively models statistical distributions of signal envelopes and enables discrimination of IEDs from background EEG (for details and code, see Janca et al., 2015). Channels exhibiting IEDs rate higher than 6.5 IEDs/minute (above the algorithm’s false positive rate) were excluded (mean = 5.0, SD = 5.1 channels per participant, Supplementary Table 1).

#### Channel Selection

A significant number of human studies have explored the cerebral correlates of motor memory acquisition and consolidation. These studies have highlighted the importance of the frontal, cingulate and parietal cortices, the striatum, hippocampus and cerebellum in learning and subsequent consolidation of newly acquired motor sequence (Walker et al., 2005; Albouy et al., 2008, 2013; Debas et al., 2014; Doyon et al., 2018). Accordingly, channels which were assigned to one of these brain regions were defined as being part of the “motor-learning network.”

### Gamma-Band Envelope Estimation

The instantaneous signal amplitude in the gamma-band was computed using the Hilbert Transform. Continuous EEG signals were first bandpass filtered in multiple successive 10 Hz wide frequency bands between 60 and 140 Hz (or between 60 and 100 Hz for data with a sampling rate of 250 Hz, *N* = 2 participants) using zero-phase forward and reverse Finite Impulse Response filters (FIR, order = 3 cycles of the low frequency cut-off, implemented in Fieldtrip). Next, for each bandpass filtered signal, the envelope signal (analytic amplitude) was (i) computed using the standard Hilbert transform (implemented in Fieldtrip), (ii) smoothed using a 0.03 s moving average temporal smoothing window, and (iii) normalized to the rest session (i.e. the mean and the standard deviation from the rest session were used to normalize (*z*-score) the entire data-set, including the rest session). Finally, the normalized and smoothed envelope signals per frequency band were averaged together to provide one single time series (the high gamma-band envelope) across the entire data-set. This approach, as the broadband gamma selection, is inspired by numerous previous studies (Swann et al., 2009; Ossandón et al., 2011; Vidal et al., 2012; Combrisson et al., 2017).

### Feature Extraction for Classification

#### Data Epoching

Data were epoched using a time-window of 0.3 s. Data during the finger tapping task were epoched in 0.3 s windows around each correct button press. Data from the ∼4 min-long rest session was epoched into consecutive (non-overlapping) 0.3 s-long time-windows, and data from each 30 s-long sleep epoch was epoched into consecutive (half-overlapping) 0.3 s-long time-windows. Trials exhibiting values > 10 a.u. were excluded. On average (± SD), the percentage of rejected 0.3 s long trials was 1.87% (±2.07), 1.43% (±1.06), 6.18% (±4.80), and 7.33% (±7.07), during the task, rest, sleepPre and sleepPost sessions, respectively.

#### Feature Extraction

Each 0.3 s-long epoch corresponded to one “trial” (also known as “observation”) for classification. The mean gamma amplitude per electrode was computed resulting in N features per trial (where N is the number of electrodes). Trials from the learning/retest sessions were labeled as “motor-class.”. Trials from the rest session were labeled as “rest-class.” Trials from the two sleep periods were designated as “unlabeled.”

For each participant, the final data-set contained the same number of motor- and rest-trials. This number was determined by the class having fewer trials (random selection when required). In 1 participant (P#1), the trials during retest were excluded due to excessive artifacts during that period. The total number of sleep-trials was dependent on the number of 30 s-epochs scored in NREM sleep during the 2 sleep periods, respectively.

### Support Vector Machine Approach

We implemented support vector machine (SVM) binary classification algorithms (Boser et al., 1992; Cortes and Vapnik, 1995) using Matlab (Statistics and Machine Learning Toolbox). The SVM training algorithm searches for an optimal hyperplane that maximizes the margins between the nearest features of different classes. Different kernel methods can be used, such as linear, radial basis function or polynomial kernels. The classification approach was performed in 2 consecutive steps schematized in Figure 1B. Please note that we also implemented Linear Discriminant Analysis classification algorithms that confirmed the results we observed with SVM.

#### Step1: Train an SVM Model Using Wake Data and Cross-Validate the Model

An SVM model was built using the labeled waking data (“motor” and “rest”-trials). We used a linear kernel in the function fitscvm implemented in Matlab (parameters: “KernelFunction,” “linear”; “Standardize,” true; “KernelScale,” 1).

Classification performance was calculated using 5-fold cross-validation. The data set (i.e. “motor-” vs. “rest-trials”) was first randomly partitioned into 5 equal sized subsamples. Then, of the five subsamples, four subsamples were used as training data while the remaining subsample was retained as validation data. The cross-validation process was repeated five times, with each of the subsamples used once as the validation data. Each of the five trained classifiers was then applied on its corresponding validation data, and its predictive accuracy computed (i.e. the percentage of trials correctly classified). Finally, the mean of the five predictive accuracies was calculated and defined as the decoding accuracy (DA). The functions crossval followed by kfoldLoss implemented in Matlab were used to cross-validate the model.

#### Step 2: Apply the Validated SVM Model on Sleep Data

The SVM model (from step1) was used to predict the class labels (“motor” or “rest”) of the unlabeled “sleep”-trials using Matlab function predict. For each sleep epoch scored as NREM sleep, a label was assigned to each of its accepted “sleep”-trials (0.3 s-long each, 50% overlap, see data epoching above). Finally, the proportion of motor-votes was computed per 30 s-long NREM epochs and in respect to the sleep period (sleepPre vs. sleepPost).

### Statistical Analysis

SPSS software (v22, IBM SPSS Statistics, Armonk, NY, United States) and Matlab were used to perform statistical analysis. Memory performance extracted from blocks 2:3, 9:10, and 12:13 were compared using *repeated measures ANOVA* (two-tailed, *p* < 0.05, Greenhouse-Geisser correction), and post-hoc comparisons using *paired-sample t-test* (two-tailed, *p* < 0.05).

For each participant, the proportions of motor-votes for sleepPre and sleepPost were compared using a *Wilcoxon rank sum test* (left-tailed since we hypothesized an increase in the proportion of motor-votes during sleepPost in comparison with sleepPre, *p* < 0.025). The effect size *r* of the test was computed by dividing the z-*statistic* value by the square root of the total number of observations.

At the group level, the median proportion of motor-votes during sleepPre and sleepPost were compared using a *paired-sample t-test* (two-tailed, *p* < 0.05). The correlation between the level of reactivation and memory performance was assessed using *Pearson’s r* (two-tailed, *p* < 0.05).

## Results

### Motor Memory Performance

**FIGURE 2:**
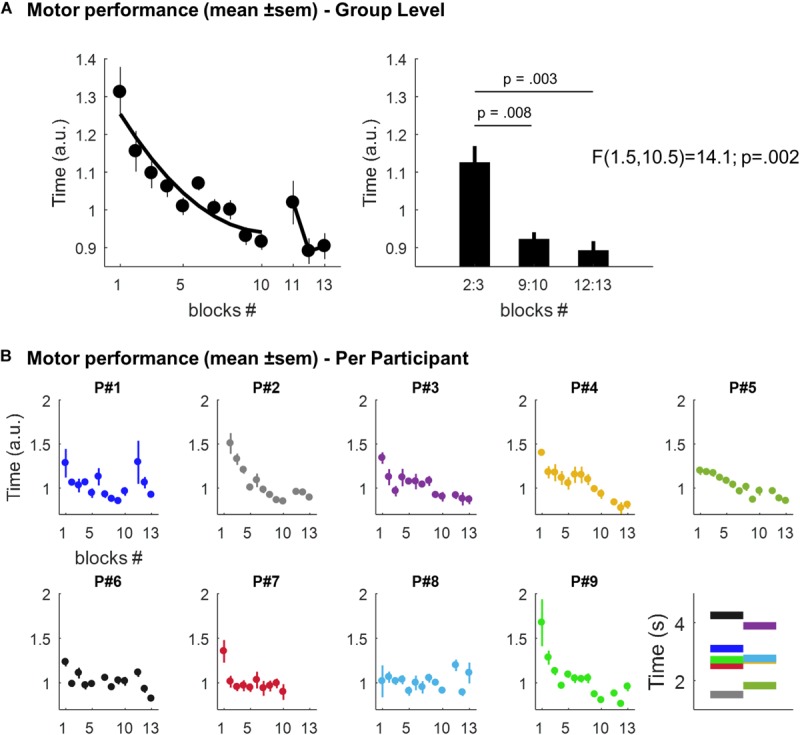
**(A)** Motor performance at the group level. Left panel: scatter plots (dots) and best second degree polynomial fit (black line) of the mean time (± SEM; arbitrary units, a.u.) to play the 6-digit sequence across the 10-blocks of the learning session and the 3-blocks of the retest, respectively. Right panel: mean time (± SEM; a.u.) to play the sequence in blocks 2:3, 9:10, and 12:13, respectively. **(B)** Individual motor performances. Scatter plots (dots) of the mean time (± SEM; a.u.) to play the 6-digit sequence across blocks for each participant. The last panel displays, for each participant, the mean performance (in seconds) across all the sequences (mean = 2.81, range: 1.52–4.25). Per participant, each sequence’s repetition was divided by this mean value. This yields performance values expressed relative to a “baseline level”, thus facilitating comparison between participants. Note that motor performance in Participant P#2 was not record during the first block for technical reasons and P#7 declined to run the retest session at the end of the nap session.

The mean time to play the 6-digit sequence across the 10-blocks of the learning session and the 3-blocks of the retest is displayed in Figure 2A (at the group level) and in Figure 2B (individual performances). Overall, we observed time-dependent improvements in performance across the course of the learning session i.e. participants were getting faster across trials as reflected by a decrease in the time to play the sequence. There was a statistically significant effect of trials (i.e. blocks 2:3, 9:10, and 12:13, respectively), on memory performance [*F*(1.5,10.5) = 14.1; *p* = 0.002]. During the learning session, performance improved by 18.04% [mean = 1.125, SEM = 0.044 a.u. in blocks 2:3, and mean = 0.922, SEM = 0.019 in blocks 9:10; *t*(8) = 3.5, *p* = 0.008]. This improvement was preserved after the nap session [mean = 0.892, SEM = 0.025 in blocks 12:13; *t*(7) = 4.3, *p* = 0.003 in comparison with blocks 2:3], but no significant improvement was observed in comparison with blocks 9:10 [*t*(7) = 0.8, *p* = 0.453].

### Electrodes Localization

**FIGURE 3:**
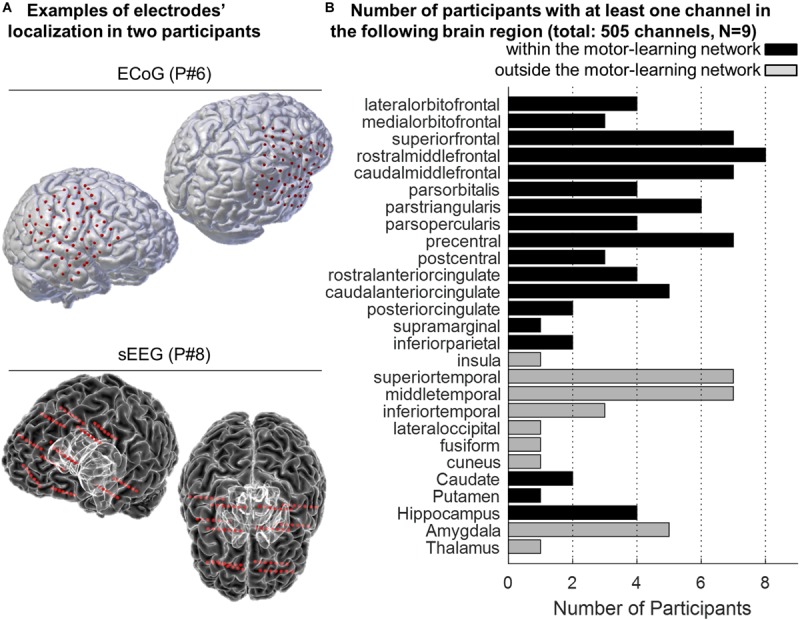
**(A)** Examples of 3-D reconstruction of the brain of two participants implanted with ECoG (P#6, top panel) and sEEG (P#8, bottom panel). Red dots represent the localization of the electrodes onto the participants’ pial surface. **(B)** Anatomical distribution of the electrodes across participants. The histogram displays the number of participants with at least one electrode in the different targeted brain areas. The “motor-learning network” includes the frontal, cingulate and parietal cortices, the striatum and the hippocampus (in total 372 electrodes; no electrode targeted the cerebellum).

In total, 550 channels were initially considered (mean = 61.1, SD = 16.8), 505 were analyzed (mean = 56.1, SD = 16.5), and 372 (mean = 41.3, SD = 13.3) were identified as being part of the motor-learning network (Supplementary Table 1 and Figure 3).

### Sleep Periods

The mean (±SD) sleep period time (SPT, from the first to the last sleep epoch) in the nap following the rest/learning sessions (i.e. “sleepPost”) was 38.4 ± 16.1 min (see Supplementary Table 2). The sleep period was predominated by NREM sleep (34.3 ± 14.1 min, 88.3 ± 10.0% of SPT). The mean sleep period time in the control “sleepPre” was 51.8 ± 27.4, including 42.8 ± 20.3 NREM sleep (85.1 ± 12.4% of SPT).

**FIGURE 4:**
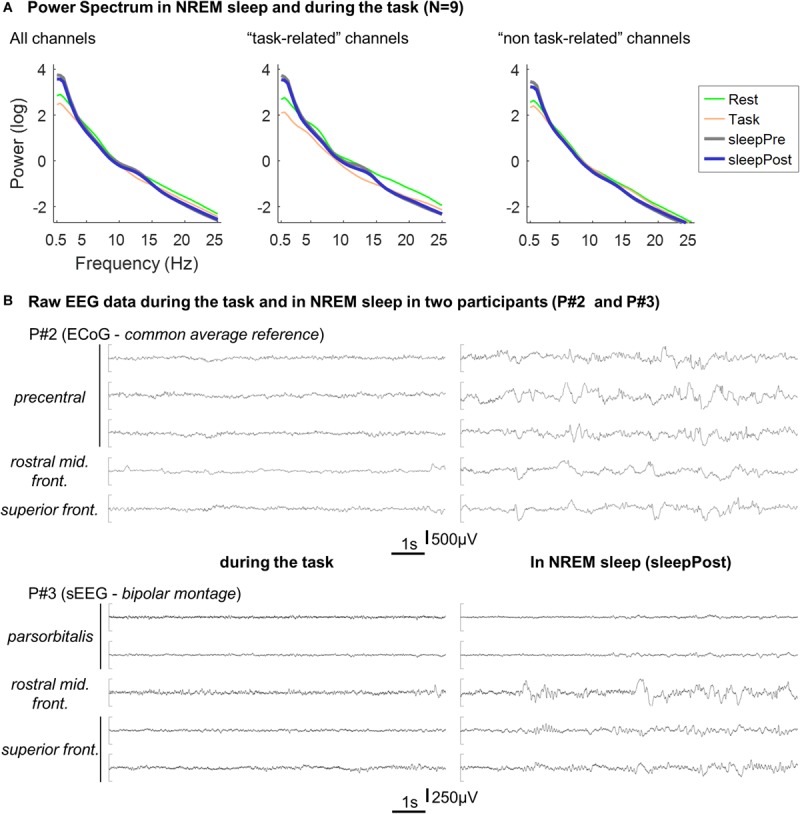
**(A)** Averaged Power spectrum in NREM sleep and during the task. Per electrode, the power spectrum was calculated for each 30 s-long NREM sleep epochs (“sleepPre” and “sleepPost”), for each block of the tapping task (“Task”) and for each consecutive 30 s-long windows in rest (“Rest”) using Fieldtrip’s ft_freqanalysis function (method: “mtmfft,” taper: “dpss,” from 0.5 to 25 Hz in steps of 0.5 Hz). Per participant and condition (sleepPre, sleepPost, task and rest), power spectrum was then averaged across epochs and across electrodes and log-transformed before being finally averaged across participants. The procedure described above was performed using all the channels, and using the five channels exhibiting the highest vs. lowest linear predictor coefficients β. Since the larger is |βj|, the more important is the contribution of the jth feature (i.e. channel) in the decision function (e.g. Chang and Lin, 2008), this approach allowed to compare channels that contributed the most to the motor-task (e.g. precentral gyrus, middle frontal gyri) from channels that contributed the least (e.g. superior and middle temporal gyri). **(B)** Example of EEG recordings in two participants. Ten seconds-long segments of EEG recording during the task (left panel) and in NREM sleep (right panel) in two participants (P#2 and P#3). The displayed channels are the channels exhibiting the highest linear predictor coefficients |β|.

Power spectra of the two sleep periods were computed and are displayed in Figure 4. In comparison with the spectrum during the task, the two sleep EEG spectra exhibit a clear increase in slow-frequencies (below 4 Hz). In addition, a relative increase can be seen in the spindle-band, especially using the “task-related” channels (see legend). The two sleep EEG spectra (using all the channels) did not show any significant difference (two-way repeated measure ANOVA; sleep effect: *F*(1,8) = 1.1, *p* = 0.34).

### Neural Classification and Decoding Accuracy During the Task

**FIGURE 5:**
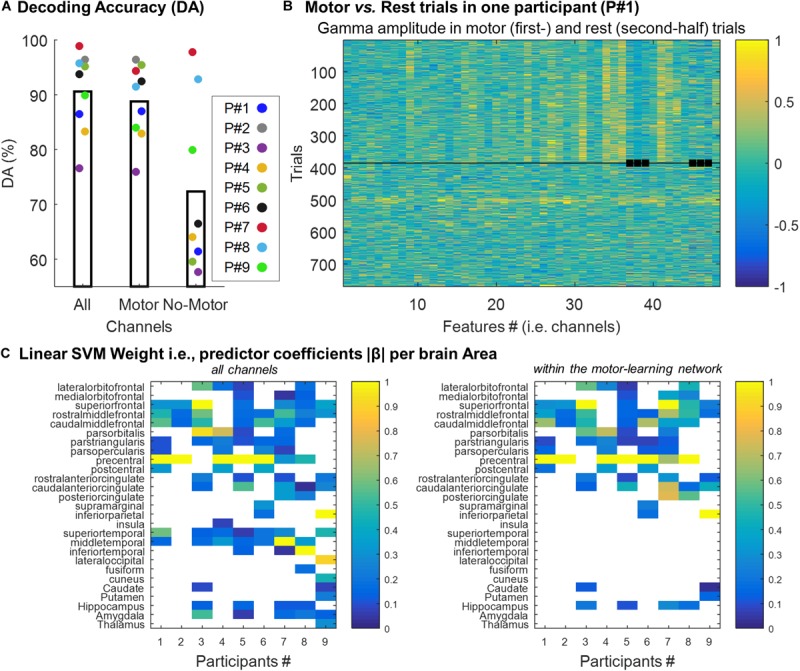
**(A)** Individual decoding accuracy achieved by the classifier (mean across all folds per participant) using all the channels (“All,” 505 channels), and using the channels within (“Motor,” 372 channels) and outside (“No-Motor,” 133 channels) the motor-learning network, respectively. **(B)** Example of gamma-band activity (Participant #1) used as predictors to which SVM classifier was trained. Each row corresponds to one 0.3 s-long trial (i.e. observation), and each column corresponds to one feature (i.e. channel). The squares point-out features/channels outside the motor-learning network. The horizontal line separates the motor-trials (first-half) from the rest-trials (second-half). **(C)** Linear predictor coefficient β across brain areas. Per participant and brain area, the absolute β (rescaled [0–1]) value was extracted. It allowed exploring the contribution of the different brain areas in the decision function. The analysis was performed using the two set of channels (all the channels vs. the channels within the motor-learning network), separately.

We trained a support vector machine (SVM) binary classification model to discriminate between “motor” vs. “rest” trials. The decoding accuracy (DA) achieved by the classifier was computed using standard 5-fold cross-validation (see section “Materials and Methods”). DA was computed using all the channels (505 channels in total), and using the channels within (372 channels) and outside (133 channels) the motor-learning network. The mean DA across participants was 90.6% (SD = 7.3; min = 76.5, max = 98.8), 88.8% (SD = 6.9; min = 75.8, max = 96.3) and 72.3% (SD = 15.7; min = 57.5, max = 97.7; 5 participants below 70%) using all the channels, the channels within- and the channels outside- the motor-learning network, respectively (Figure 5A). Figure 5B illustrates “motor” vs. “rest” trials used for classification in one participant.

To assess the contribution of the different brain areas in the decision function, the linear predictor coefficients β were analyzed as a function of the anatomical localization of the channels. The larger is |βj|, the more important is the contribution of the jth feature in the decision function (e.g. Chang and Lin, 2008). After being rescaled from 0 to 1, |β| was extracted for each brain area. When more than 1 feature (i.e. channel) was localized in a given brain area, the largest |β| was used. The analysis was done across participants, and for the two sets of channels (all the channels vs. the channels within the motor-learning network) separately (Figure 5C). Not surprisingly, this analysis highlighted the importance of the channels implanted in the precentral gyrus in the decision function. Over the seven participants having at least 1 channel in the precentral gyrus, 5 (71%) and six participants (86%) exhibited the largest |β| from this area when, respectively, all the channels (left panel) and the channels from the motor-learning network (right panel) were considered. Nevertheless, this analysis also revealed the relative importance of other brain areas, mainly from the frontal lobe, in the classification decision, while the contribution of medial temporal lobe structures were, in comparison, relatively low.

### Proportion of Sleep-Windows Classified as Motor- vs. Rest-Classes During Sleep and Correlation With Performance Change

We hypothesized that the gamma-band pattern underlying finger-movements’ from rest re-occurred more often during sleep following learning than during an earlier control sleep period. After having trained and cross-validated a support vector machine (SVM) model (binary classification i.e. “motor” vs. “rest”), we applied the trained model on the “unlabeled” NREM sleep data immediately after the task as well as during a control sleep period preceding the task.

**FIGURE 6:**
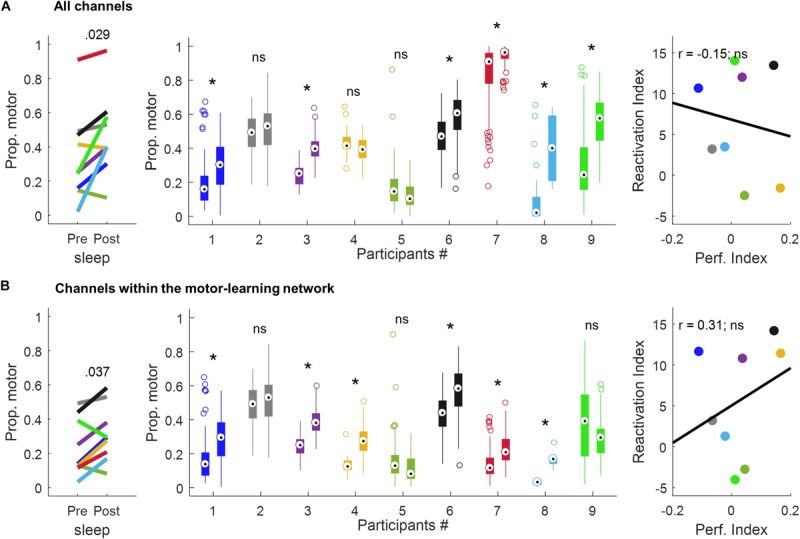
Proportion of motor votes during sleep and correlation with performance improvement using all the channels **(A)** and the channels within the motor-learning network **(B)**. The left-panel displays the median proportion of motor votes during sleepPre and sleepPost, respectively. The *p*-value is of a *paired-sample t-test* (two-tailed, *p* < 0.05). The middle-panel displays, for each participant and sleep period, boxplots of the proportion of motor-votes within 30 s-long sleepPre and sleepPost NREM sleep epochs (*Wilcoxon rank sum test*, left-tailed, *p < 0.025). The right-panel displays the correlation between the reactivation index (i.e. difference in the median proportion of motor-votes [Post-Pre] multiplied by the number of sleep epochs in sleepPost) and the performance index (i.e. difference between blocks 9,10 of learning and blocks 12,13 of the retest [blocks9,10-blocks12,13]; *Pearson’s r*, two-tailed, *p* < 0.05). ns, non-significant.

Using all the channels (Figure 6A and Table 1), a significant increase in the proportion of motor-votes during sleepPost was observed in six participants (Ps#1, 3, 6, 7, 8, and 9). At the group level, the comparison of the median values between the 2 sleep periods (paired-sample *t*-test, two-tailed, *p* < 0.05) revealed a significant difference [*t*(8) = −2.66; *p* = 0.029]. There was no significant correlation between the reactivation index (i.e. difference in the median proportion of motor-votes [Post-Pre] multiplied by the number of sleep epochs in sleepPost) and the performance index (i.e. difference between blocks 9, 10 of learning and blocks 12, 13 of the retest [blocks9,10-blocks12,13]; Pearson’*r* = −0.15, *p* = 0.73).

**TABLE 1:** Median (± SD) proportions of motor-votes during sleep before (sleepPre) and after (sleepPost) the learning task, and their comparison (z-*statistic*, *p*-value and effect size *r* of a Wilcoxon rank sum test, left-tailed, *p* < 0.025) using all the channels and the channels within the motor-learning network, respectively.

**P#**	**All channels**					**Channels within the motor-learning network**				
	**sleepPre**	**sleepPost**	***z***	***p***	***r***	**sleepPre**	**sleepPost**	***z***	***p***	***r***
1	0.16 ± 0.14	0.30 ± 0.15	−4.64	1.73e-06	−0.36	0.14 ± 0.14	0.29 ± 0.15	−4.91	4.54e-07	−0.38
2	0.49 ± 0.13	0.53 ± 0.15	−0.87	0.19	−0.07	0.49 ± 0.13	0.53 ± 0.15	−0.87	0.19	−0.07
3	0.25 ± 0.06	0.40 ± 0.08	−8.42	1.82e-17	−0.74	0.25 ± 0.06	0.38 ± 0.09	−7.76	4.23e-15	−0.68
4	0.41 ± 0.08	0.39 ± 0.08	2.31	0.99	0.22	0.12 ± 0.05	0.27 ± 0.09	−7.48	3.73e-14	−0.70
5	0.15 ± 0.12	0.10 ± 0.08	2.65	1.00	0.22	0.13 ± 0.12	0.08 ± 0.08	2.60	1.00	0.21
6	0.47 ± 0.11	0.61 ± 0.13	−7.24	2.18e-13	−0.46	0.44 ± 0.10	0.58 ± 0.14	−7.72	5.77e-15	−0.49
7	0.91 ± 0.17	0.96 ± 0.06	−5.10	1.71e-07	−0.34	0.12 ± 0.08	0.21 ± 0.09	−7.46	4.38e-14	−0.50
8	0.02 ± 0.15	0.40 ± 0.19	−3.94	4.14e-05	−0.55	0.03 ± 0.02	0.17 ± 0.04	−4.66	1.59e-06	−0.65
9	0.24 ± 0.20	0.58 ± 0.17	−6.44	5.95e-11	−0.52	0.39 ± 0.23	0.29 ± 0.12	1.88	0.97	0.15

Using the channels within the motor-learning network (Figures 6B and Table 1, see also Supplementary Figure 1), a significant increase in the proportion of motor-votes during sleepPost was observed in six participants (Ps#1, 3, 4, 6, 7 and 8). At the group level, the comparison of the median values between the two sleep periods (paired-sample *t*-test, two-tailed, *p* < 0.05) revealed a significant difference [*t*(8) = −2.50; *p* = 0.037]. There was no significant correlation between the reactivation index and the performance index (Pearson’*r* = 0.31, *p* = 0.46).

## Discussion

In this study, we employed machine learning methods (SVM) applied to intracranial EEG (iEEG) signals to investigate spontaneous motor-related reactivation of neural patterns in human sleep. This approach allowed identification, at the single-subject level, of the reactivation of gamma-band patterns in NREM sleep that were first identified in wake during a motor task. This study provides electrophysiological evidence for the reactivation in human sleep of neural activity related to prior waking experience, i.e. that a central tenet of the reactivation theory does occur in human sleep.

### Electrophysiological Evidence of Motor-Related Reactivation Events in Human Sleep

In humans, several non-invasive functional neuroimaging (TEP, fMRI) studies have provided evidence of spontaneous (Maquet et al., 2000; Peigneux et al., 2004; Deuker et al., 2013) or triggered (Rasch et al., 2007; van Dongen et al., 2012) reactivation of learning-related cerebral activity during sleep. In addition, recent electroencephalography (EEG) studies provided more direct, electrophysiological evidence of spontaneous reactivation in humans by showing that sleep scalp EEG contains features specific to the task previously experienced (Schönauer et al., 2017), and by showing, using intracranial EEG (iEEG), the reactivation in NREM sleep of spatiotemporal sequences of gamma activity peaks that were identified in wake (Jiang et al., 2017), and the reactivation in wake and NREM sleep of item-specific gamma activation patterns that were initially identified in a memory task (Zhang et al., 2018). However, electrophysiological evidence of spontaneous reactivation in humans is still sparsely documented, and the current study importantly extends this research area by showing that the gamma-band patterns that distinguished motor from non-motor periods during a learning task were more prevalent during the sleep that followed learning compared to a control sleep period that took place before the task. This relative increase in the amount of motor-related events shows that the iEEG activity patterns recorded during motor learning were spontaneously reactivated (i.e. recapitulated) in NREM sleep. A recent scalp EEG study identified triggered motor-related reactivation during sleep using a classifier approach (Belal et al., 2018). The re-exposure of auditory cues (i.e. sounds presented as context during prior motor learning) during sleep was associated with an increase in the correct classification rate as identified by applying trained classifiers on the corresponding EEG data (Belal et al., 2018). Here, we expand the use of classifiers in the detection of spontaneous iEEG reactivation during sleep, and using gamma-band activity as a proxy of motor-information processing.

### Reactivation in NREM Sleep and Memory Function of Gamma-Band Activity

Our results support the hypothesis that NREM sleep plays a role in the processing of motor skills (Walker et al., 2002; Albouy et al., 2008; Antony et al., 2012), and emphasize the involvement of gamma activity in these NREM sleep-dependent mechanisms. While high-frequency neural activity is generally related to complex cognitive functions in wakefulness (Lachaux et al., 2012), our results suggest a link between such high-frequency activity in human NREM sleep and the ongoing processing of recently experienced events. It suggests that gamma-band activity during sleep might complement its critical role in memory encoding and retrieval in wakefulness (Johnson and Knight, 2015), by supporting the re-processing of recent experiences in NREM sleep through reactivation mechanisms. However, we did not observe any significant correlation between the level of reactivation and performance change over the rest period, and thus could not confirm that the gamma-band reactivation observed in the current study actually strengthened or stabilized the motor memory representation, i.e. is of functional significance. Other work did not find a link between neural reactivation and motor performance in humans (Belal et al., 2018), and as pointed out by the authors, “*it is noteworthy that the vast majority of the rodent work on reactivation bears no reference to behavioral consolidation*” (p.212; Belal et al., 2018). Furthermore, memory consolidation is an umbrella notion that covers multiple types of memory processes (for a review, see Stickgold and Walker, 2013). Accordingly, it is possible that the gamma-band pattern reactivation participated in subtle offline processing that was not measured in the current study. It is also possible that reactivation as reported here is not of functional significance and thus does not relate to consolidation mechanisms. The relatively low number of participants might also account for the lack of relationship to memory consolidation, and future studies are needed to clarify the link between gamma-band motor reactivation during sleep and subsequent performance changes in humans. Regardless, our results show that gamma-band activity during sleep was influenced by the motor task, and thus highlights the role of such high-frequency activity in the reactivation of recent waking experiences by the sleeping brain.

### Methodological Considerations and Future Directions

A large number of motor patterns were identified by the classifier during the pre-learning control sleep, prior to the participants having performed the experimental motor learning task. In addition, a large inter-subject variability was observed in the detection rate of motor patterns during both sleep periods. The production of sequential motor movements is a central human behavior that is omnipresent in our daily life (e.g. typing on a computer keyboard or on a cellphone). These behaviors might evoke activity during sleep which would be classified as motor-related during both sleep periods. Furthermore, multiple subject-dependent parameters such as the electrode locations or type, determined to what extent models draw strict boundaries between different activities, including within the motor activities, and consequently, to what extent they distinguish between task and non-task related memory events during sleep. Together, these parameters could account for the proportion of sleep-windows classified as motor-class during pre-learning sleep, but also for the large inter-subject variability in the detection rate of motor patterns. Accordingly, this within-subject control sleep period before the task allowed separating task-related from non-task related reactivation events, and then allowed testing the reactivation of the task-related brain activity during sleep following the task. Importantly, to test a potential effect of the time elapsed between the two sleep periods on the results, the correlation between the difference in the median proportion of motor votes between sleepPost and sleepPre and the time elapsed (in minutes) between the two sleep periods was tested using *Pearson’s r* (two-tailed, *p* < 0.05). The analysis showed no significant correlation using both all the channels (Pearson’*r* = −0.14, *p* = 0.71) and the channels within the motor-learning network (Pearson’*r* = 0.31, *p* = 0.42), suggesting that the increase in the proportion of motor-votes during sleep following the task was not dependent on the time elapsed between the two sleep periods.

The present findings show that the study of spontaneous reactivation of gamma-band patterns related to a motor learning task is feasible in human NREM sleep and by using a machine learning framework. In accordance with studies in rodents showing “true” replay that is the recurrence of sequential firing patterns during sleep (e.g. Lee and Wilson, 2002; Ramanathan et al., 2015), future studies should investigate the feasibility of using multiclass classifiers to discriminate individual finger movements that would allow testing the reactivation of the exact sequence of digits in human sleep. In addition, and since different grapho-elements in NREM sleep (i.e. slow-oscillations and spindles) appear to be linked to replay events in rodent sleep (e.g. Peyrache et al., 2009; Gulati et al., 2014; Ramanathan et al., 2015), it would be informative in the future to explore the relationship between these reactivation events and NREM oscillations in human sleep.

## Conclusion

The results reported here provide electrophysiological support for the hypothesis that reactivation of neural patterns during sleep underlies the reprocessing of memories from recent waking experiences in humans. Gamma-band patterns characterizing motor learning were found to recur more often in a subsequent nap, as compared to comparable sleep periods preceding learning.

## Data Availability Statement

The data and custom Matlab analysis scripts are available upon reasonable request from J-BE, jb.eichenlaub@gmail.com.

## Ethics Statement

The studies involving human participants were reviewed and approved by the local Institutional Review Board (Partners IRB). The patients/participants provided their written informed consent to participate in this study.

## Author Contributions

J-BE, EH, and SC designed the research. J-BE, AG, JL, and SC performed the research. J-BE analyzed the data. SB, NP, NR, MW, EH, and SC assisted in the data analysis and interpretation. J-BE, SB, NP, NR, MW, EH, and SC wrote the manuscript.

## Conflict of Interest

The authors declare that the research was conducted in the absence of any commercial or financial relationships that could be construed as a potential conflict of interest.

## Footnotes

1 http://surfer.nmr.mgh.harvard.edu

2 https://github.com/pelednoam/ieil

3 https://github.com/gpiantoni/phypno
