# Supplementary material for: Reactivation of Motor-Related Gamma Activity in Human NREM Sleep
Source: Front Neurosci. 2020 May 12;14:449. doi: 10.3389/fnins.2020.00449 (PMC7235414; doi:10.3389/fnins.2020.00449)
Supplement: Supplementary file 1 [file Data_Sheet_1.PDF]

Supplementary Material

1 Supplementary Figures and Tables

1.1 Supplementary Figures

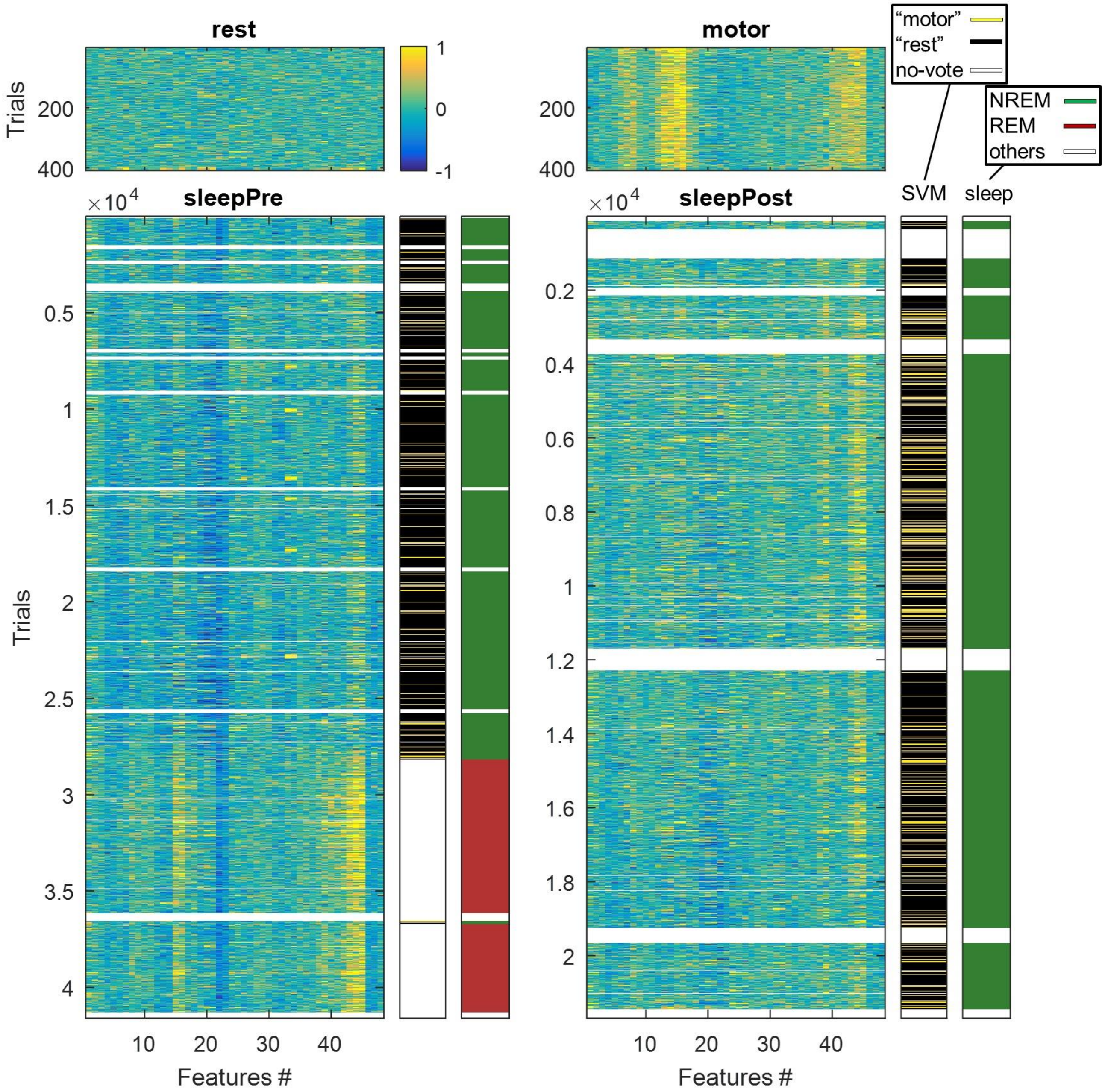

Supplementary Figure 1. Example of gamma-band activity (using channels from the motor-learning network; Participant #7) in rest, during the task and during sleepPre and sleepPost, respectively. Each row corresponds to one 0.3s-long trial and each column corresponds to one feature (i.e., channel, 48

channels in this participant). For each sleep period, the label assigned to each accepted 0.3s-long trial in NREM sleep is shown.

1.2 Supplementary Tables

P#	Sex	Age	Hand in task	EEG method	sampling rate, Hz	Number of electrodes initial	after auto rej.	motor-network
**1**	M	43	right	ECoG	2000	58	48	42
**2**	M	17	right	ECoG	250	26	26	26
**3**	F	59	right	sEEG	2000	69	60	46
**4**	M	34	right	ECoG	250	44	44	22
**5**	F	27	left	sEEG	2000	81	81	63
**6**	F	51	left	ECoG	2000	60	48	31
**7**	F	20	left	sEEG	2000	70	65	48
**8**	M	40	both*	sEEG	2000	72	72	54
**9**	M	21	right	sEEG	2000	70	61	40
Sum						*550*	*505*	*372*
Mean		*34.7*				*61.1*	*56.1*	*41.3*
SD		*14.7*				*16.8*	*16.5*	*13.3*

Supplementary Table 1. Sex: Male (M) or Female (F). Age (years). Hand used during the finger tapping task: right and/or left; *P#8 used left hand for button presses 1/2 and right hand for 3/4. Intracranial EEG methods: Electrocorticography (ECoG) or stereotactic-EEG (sEEG). Sampling rate during data acquisition (Hz). Number of electrodes *1)* initially considered (i.e. functioning; “initial”), *2)* after automatic detection/rejection of pathological channels (“after auto rej.”) and *3)* identified as being part of the motor-learning network (“motor-network”; see Materials and Methods for details), respectively.

P#	sleep	day	time	SPT	NREM		REM		Wake		Ind/Mvt	
				min	min	%	min	%	min	%	min	%
**1**	pre	3	2:18:32	47.5	46.0	96.8	-	-	-	-	1.5	3.2
	post	3	13:21:54	40.0	37.0	92.5	-	-	-	-	3.0	7.5
**2**	pre	6	9:53:20	32.5	32.0	98.5	-	-	-	-	0.5	1.5
	post	6	14:25:20	44.5	41.5	93.3	-	-	1.0	2.2	2.0	4.5
**3**	pre	5	11:21:42	33.5	24.5	73.1	-	-	4.0	11.9	5.0	14.9
	post	5	12:59:42	55.5	41.0	73.9	-	-	6.5	11.7	8.0	14.4
**4**	pre	4	9:14:55	21.0	19.5	92.9	-	-	-	-	1.5	7.1
	post	4	16:25:55	40.0	38.0	95.0	-	-	-	-	2.0	5.0
**5**	pre	7	6:03:09	60.0	44.0	73.3	11.5	19.2	-	-	4.5	7.5
	post	7	12:03:22	33.0	29.5	89.4	2.0	6.1	-	-	1.5	4.5
**6**	pre	2	10:06:36	79.5	75.5	95.0	-	-	-	-	4.0	5.0
	post	3	14:39:13	51.5	49.0	95.1	-	-	-	-	2.5	4.9
**7**	pre	3	23:40:30	103.5	66.0	63.8	31.5	30.4	1.0	1.0	5.0	4.8
	post	4	14:31:10	53.5	47.5	88.8	-	-	3.5	6.5	2.5	4.7
**8**	pre	3	10:26:02	25.5	21.0	82.4	-	-	0.5	2.0	4.0	15.7
	post	3	13:48:57	6.5	4.5	69.2	-	-	-	-	2.0	30.8
**9**	pre	3	6:29:42	63.5	57.0	89.8	-	-	0.5	0.8	6.0	9.4
	post	3	11:35:49	21.5	21.0	97.7	-	-	-	-	0.5	2.3
	pre		*Mean*	51.8	42.8	85.1	21.5	24.8	1.5	3.9	3.6	7.7
			SD	27.4	20.3	12.4	14.1	8.0	1.7	5.4	1.9	4.9
	post		*Mean*	38.4	34.3	88.3	2.0	6.1	3.7	6.8	2.7	8.7
			SD	16.1	14.1	10.0	-	-	2.8	4.7	2.1	9.0

Supplementary Table 2. Sleep: Pre- and post-learning sleep, respectively. Day: number of days after the implantation surgery (surgery's day being day0). Time: start time of the sleep period (in the format hour:minute:second; 24-hour clock). Sleep Period Time (SPT): duration (in minutes) from the first to the last epoch of sleep (NREM or REM sleep). NREM/REM sleep: duration (in minutes) and percentage (in SPT) of NREM and REM sleep, respectively. Wake: duration (in minutes) and percentage (in SPT) of intra-sleep wakefulness. Indeterminate/movements (Ind/Mvt): duration (in minutes) and percentage (in SPT) of indeterminate or movements (e.g., transition between sleep-stages).
